# The Role of Hydraulic Silicate Cements on Long-Term Properties and Biocompatibility of Partial Pulpotomy in Permanent Teeth

**DOI:** 10.3390/ma14020305

**Published:** 2021-01-08

**Authors:** Chung-Min Kang, Saemi Seong, Je Seon Song, Yooseok Shin

**Affiliations:** 1 Department of Pediatric Dentistry, College of Dentistry, Yonsei University, Seoul 03722, Korea; kangcm@yuhs.ac (C.-M.K.); songjs@yuhs.ac (J.S.S.); 2 Department of Conservative Dentistry and Oral Science Research Center, College of Dentistry, Yonsei University, Seoul 03722, Korea; sam@yuhs.ac

**Keywords:** hydraulic silicate cements, mineral trioxide aggregate, partial pulpotomy, biocompatibility

## Abstract

The use of hydraulic silicate cements (HSCs) for vital pulp therapy has been found to release calcium and hydroxyl ions promoting pulp tissue healing and mineralized tissue formation. The present study investigated whether HSCs such as mineral trioxide aggregate (MTA) affect their biological and antimicrobial properties when used as long-term pulp protection materials. The effect of variables on treatment outcomes of three HSCs (ProRoot MTA, OrthoMTA, and RetroMTA) was evaluated clinically and radiographically over a 48–78 month follow-up period. Survival analysis was performed using Kaplan–Meier survival curves. Fisher’s exact test and Cox regression analysis were used to determine hazard ratios of clinical variables. The overall success rate of MTA partial pulpotomy was 89.3%; Cumulative success rates of the three HSCs were not statistically different when analyzed by Cox proportional hazard regression analysis. None of the investigated clinical variables affected success rates significantly. These HSCs showed favorable biocompatibility and antimicrobial properties in partial pulpotomy of permanent teeth in long-term follow-up, with no statistical differences between clinical factors.

## 1. Introduction

Hydraulic silicate cements (HSCs) such as mineral trioxide aggregate (MTA) have been developed to be the material of choice for vital pulp therapy due to their excellent sealing ability [1] and biocompatibility, along with dentin bridge formation in animal or human teeth [2,3]. These HSCs form calcium silicate hydrate and calcium hydroxide in contact with water. Release of calcium and hydroxyl ions promotes pulp healing and mineralized tissue formation [4] while the alkalinity contributes to antimicrobial properties [5,6]. ProRoot MTA (Dentsply Tulsa Dental, Tulsa, OK, USA) is a brand of MTA that shows good biocompatibility. M. G. Gandolfi reported that ProRoot MTA exhibited bioactive potential as it allows osteoid matrix deposition by activated osteoblasts and favors its biomineralization and achieved a direct bond between the material’s surface and the mineralized bone matrix [7]. However, it has some major drawbacks such as heavy metal composition (arsenic, chromium, and lead) [8], difficulty of handling and long setting time [9], and tooth discoloration [10]. Other bioactive cements that have been developed and used in vital pulp therapy include calcium-enriched mixture cements such as Biodentine (Septodont Inc., Saint-Maur-des-Fossés, France), Bioaggregate (Innovative Bioceramix, Vancouver, BC, Canada), MTA Angelus (Soluçoes Odontologicas, Londrina, Brazil), iRoot BP Plus (Innovative Bioceramix Inc, Vancouver, Canada) Endocem Zr (Maruchi, Wonju, Korea), and Endocem MTA (Maruchi, Wonju, Korea). OrthoMTA (BioMTA, Seoul, Korea) and RetroMTA (BioMTA, Seoul, Korea) were also developed as biocompatible materials for pulp therapy. OrthoMTA has been shown to contain fewer heavy metal components than ProRoot MTA [11] and lower expansion with excellent sealing ability [11]. In a previous study, OrthoMTA showed more cytotoxic effects than ProRoot MTA, suggesting that calcium silicate-based cements may differ in terms of the effect of ions released from the materials [12]. RetroMTA is a mixture of hydrophilic powders that is not derived from Portland cement [13]. The clinical advantage of RetroMTA is its shorter setting time (initial setting time of about 150 s) [14] and less discoloration [15]. The zirconium complex in RetroMTA contributes to its shorter setting time by modifying its physical properties and the chemistry of its setting. [12]. However, in terms of sealing ability, RetroMTA has shown the highest dye-absorbance values when compared with ProRoot MTA and Biodentine [13]. Despite the increase in the use of OrthoMTA and RetroMTA as vital pulp therapy agents, there is limited information about these cements in the literature.

According to consensus recommendations from the International Caries Consensus Collaboration, preserving pulpal health should be a primary goal in the management of deep carious lesions [13]. However, if pulp exposure is sensible and non-symptomatic (i.e., presumably healthy), minimally invasive techniques should be considered for the management of pulp exposure in vital teeth [14]. Vital pulp therapy, including direct pulp capping, partial pulpotomy, and complete pulpotomy, preserves remaining pulp tissue, allowing healing and construction of the pulp–dentin complex through continuing tooth vitality [15,16,17]. Several studies have reported highly successful clinical outcomes of partial pulpotomy with HSCs. Taha et al. have reported that in partial pulpotomy of mature permanent teeth clinically diagnosed with irreversible pulpitis, ProRoot MTA sustained a good success rate (85%) over the 2-year follow-up [18]. Moreover, in another study, ProRoot MTA showed no radiographic signs of failure or clinical symptoms over a 2-year period, although 21.4% of teeth did not respond to vitality testing [19]. Our previous study reported that partial pulpotomy with RetroMTA, OrthoMTA, and ProRoot MTA showed no significant differences in outcomes among treatments up to 1 year with the following success rates: ProRoot MTA, 96.0%; OrthoMTA, 92.8%; RetroMTA, 96.0% [20]. Although some investigators have applied survival analysis to evaluate outcomes of partial pulpotomy, there are no randomized controlled studies reporting the long-term clinical outcomes of various HSCs materials. We suggest that late failures should be analyzed to properly evaluate the effects of potential factors. The aim of this study was to evaluate the long-term prognosis and biocompatibility of partial pulpotomy in permanent teeth performed using three HSCs as capping materials.

## 2. Materials and Methods

### 2.1. Patient Selection and Inclusion/Exclusion Criteria

This study was a randomized controlled study conducted with patients who visited the Department of Conservative Dentistry and Pediatric Dentistry, Yonsei University Dental Hospital, between February 2013 and July Teeth with pulp exposure but maintaining vital pulp, as confirmed on electrical or thermal tests, were included in this study. Teeth with history of spontaneous pain, lingering pain after eating cold foods, pain after eating hot foods, and/or acute minor pain that subsided with analgesics were considered to exhibit symptomatic irreversible pulpitis and were excluded. Teeth with discomfort on percussion, vestibular swelling, and/or abnormal appearance of the periodontal ligament space on radiographs, i.e., periodontal widening or periapical radiolucency, were also excluded. Teeth with pulp exposure that occurred during caries removal or subsequent to recent trauma were included. Moreover, teeth in which hemostasis was achieved within 5 min of NaOCl use after pulp amputation were included [21]. All patients provided written informed consent before participation in the study. The study was undertaken in full accordance with institutional review board of Yonsei University Dental Hospital: Approval no. 2-2012-0053, 2-2019-The clinical trial was registered in Clinical Research Information Service (CRiS), Republic of Korea: 23 October 2015, KCT0001675.

### 2.2. Randomization and Sample Size

A single-blinded, randomized controlled clinical trial was conducted. A clinical research coordinator ensured randomization through a random number table; teeth were assigned to different treatment groups, and the practitioner was blinded to the procedure of group allocation. Teeth were assigned by the clinical coordinator to one of the following groups, wherein different types of HSCs were used: ProRoot MTA, OrthoMTA, or RetroMTA. The compositions of the three materials are as follows (Table 1) [22,23]. All investigators and participants were masked to patient distribution. This study postulated that RetroMTA and OrthoMTA treatment would not be inferior to ProRoot MTA treatment. If we compare the results of calcium hydroxide (mean 20% lower success rate than HSCs), as it has been used commonly, the success rate of the control group is more than 90%; hence, it would be reasonable to set 50% to 75% of −20%, i.e., −15% to 10%, as the non-inferiority margin. Accordingly, we calculated that 30 patients per treatment group would provide 80% power to detect a non-inferiority margin of −0.12 using a one-sided α of 0.05, assuming a 20% drop-out rate.

### 2.3. Clinical Procedure

Partial pulpotomy was conducted by several dentists under the same protocol at the Department of Conservative Dentistry and Pediatric Dentistry, Yonsei University Dental Hospital. The technique was presented in a previous article [20]. After local anesthesia (2% lidocaine with epinephrine 1:80,000; Huons, Seongnam, Korea) and rubber dam isolation, dental caries was removed using a sterile flame-shaped diamond bur (Brasseler, Savannah, GA, USA) at high speed, and a no. 6 round carbide bur (Prima Classic RA6, Prima Dental Group, Gloucester, UK) at low speed. The outermost layers of the exposed pulp tissue were removed to a depth of 1.5–2 mm using a sterile round tapered diamond bur (Komet Dental, Gebr. Brasseler, Lamgo, Germany) at high speed. The pulp wound was gently flushed with 3% sodium hypochlorite for disinfection and controlling the hemorrhage. After achieving hemostasis within 5 min, the MTA materials were assigned by the clinical coordinator. The HSCs were mixed in accordance with the manufacturer’s recommendation.

The initial setting time for ProRoot MTA was 2 h and 45 min, for OrthoMTA, 6 h, and for RetroMTA, 150 s. The ProRoot MTA and OrthoMTA groups were treated in two visits, with reinforced interim restorative material in a zinc oxide-eugenol base (for example, IRM—Dentsply Caulk, Milford, DE, USA). Within 1–2 weeks, to minimize coronal leakage, the teeth were restored using final restorative materials such as composite resin, inlays, onlays, or crowns, depending on the cavity shape and size. Teeth in the RetroMTA group were covered with resin-modified glass ionomer cement after 5 min of cement application and were restored using final restorative materials on the same day.

### 2.4. Clinical and Radiographic Evaluation

The patients were followed up to assess their clinical and radiographic signs and symptoms. Follow-ups were performed at 1, 3, 6, and 12 months and at regular check-ups (every 6 and 12 months) beyond 12 months. Clinical evaluation was performed by pediatric and endodontic specialists unrelated to this study. All periapical radiographs were taken using extension cone paralleling film positioning devices to increase the dimensional accuracy of the dental X-ray images. The clinical and radiographic failure criteria were as follows: (1) spontaneous pain (Visual Analogue Scale ≥1); (2) sensitivity to palpation or percussion; (3) pathological root resorption; (4) periapical radiolucency; (5) no response on pulp vitality tests. One or more of these symptoms was considered a failure. Periapical radiographs were evaluated under optimum viewing conditions by two experienced examiners (one endodontist and one pediatric dentist) who were blinded to the capping material. Intra-observer reliability was calculated by the Cohen kappa coefficient of agreement index. The results of the Cohen kappa statistics showed good intra-observer agreement of 0.Final analysis maintained the prognostic balance generated from the original random treatment allocation by intention-to-treat (ITT) analysis.

### 2.5. Histologic Evaluation

One case treated with ProRoot MTA was attributed to follow-up drop-out as it was extracted due to localized chronic advanced periodontitis, 78 months after treatment. This case was considered as a follow-up loss since the patient’s last visit was at 18 months before his revisit to the clinic at 78 months. The tooth was extracted and embedded in paraffin without decalcification. Serial, 5-μm thick sagittal sections were obtained, stained with hematoxylin and eosin (HE) for histological analysis, and observed under a light microscope.

### 2.6. Statistical Analysis

This study evaluated several potential prognostic factors such as age, arch, tooth type, type of HSC, cause of exposure, apex status, distance to exposure (distance between the cavity and pulp as seen in the periapical radiograph), site of exposure, and final restoration. Kaplan–Meier analysis was used for assessment of the cumulative success proportion and mean time. Fisher’s exact test was used to analyze the association between various variables. Prognostic clinical variables were also identified through a univariate Cox proportional hazard regression analysis. Hazard ratios and their respective 95 percent confidence intervals were obtained. All hypothesis tests were performed at a significance level of 0.The analyses were done using SPSS software version 25.0 (IBM SPSS Inc., Chicago, IL, USA) and R version 3.6.1 (R Foundation for Statistical Computing, Vienna, Austria).

## 3. Results

**Figure 1:**
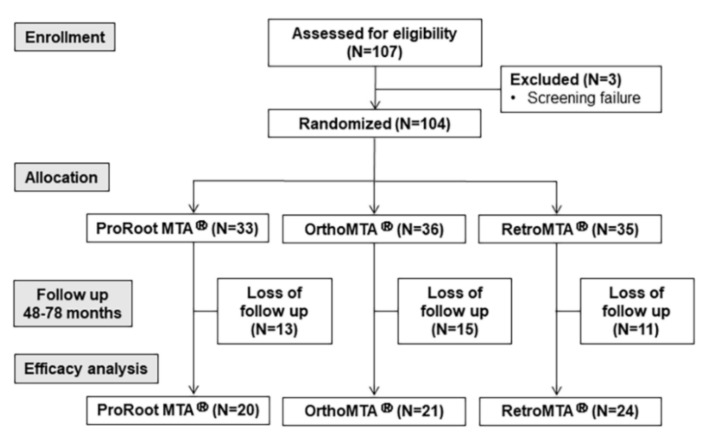
Patient flowchart of partial pulpotomy with three hydraulic silicate cements (HSCs) during a 48–78 months follow-up.

In total, 82 patients (aged 29.3 ± 14.83 years; 51 females and 31 males) were included, and 104 teeth were treated. Initially, 33, 36, and 35 teeth were initially allocated to the ProRoot MTA, OrthoMTA and RetroMTA groups, respectively. The follow-up periods were recorded as within 1 year for 78/104 teeth and as 48 to 78 months for 65/104 teeth. Twenty cases of ProRoot MTA, 21 of OrthoMTA, and 24 of RetroMTA were included in this study. The overall recall rate of more than 4 years was 62.5%, and the mean follow-up period was 62.7 months. Some patients who refused asymptomatic check-ups were unable to follow the recall schedule and were considered “drop-outs”. The progress report of partially pulpotomized cases after overall follow-up is depicted as a flowchart in Figure 1.

**Figure 2:**
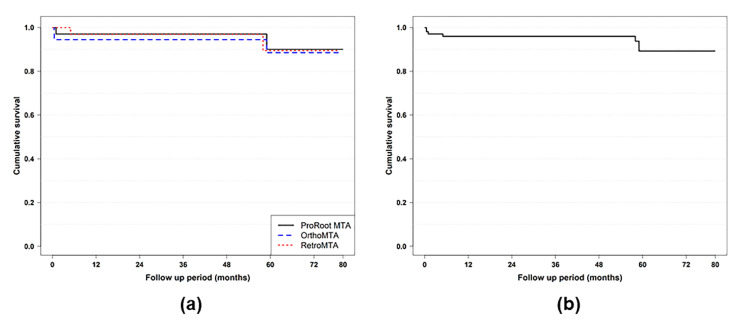
Kaplan–Meier curves of failure-free outcomes in partial pulpotomy using three hydraulic silicate cements. (**a**) The cumulative rates suggest that there was no significant difference between the three tested materials. (**b**) The overall success rate of HSC partial pulpotomy was 89.3%.

When the results were analyzed with 65 cases that have been monitored for more than 4 years, no association was found by Fisher’s exact test between success rates and age, arch, tooth type, type of HSCs, cause of exposure, apex status, distance to exposure, site of exposure, and final restoration (*p* > 0.05, Table 2). The three tested HSC materials did not show significant differences by Kaplan–Meier cumulative survival analysis (Figure 2). If all cases, including follow-up loss within 4 years, were analyzed together, the Cox proportional hazard regression analysis showed that the cumulative success rates of the three materials were not statistically different (Table 3). None of the clinical variables significantly affected the outcome of MTA partial pulpotomy when all data were combined (*p* > 0.05 for all, Table 3). Therefore, another multivariate Cox proportional hazard regression analysis to determine the decisive factor for survival rate was not performed.

During the 1 year follow-up, clinical failure happened in two OrthoMTA-treated teeth, one ProRoot MTA-treated tooth, and one RetroMTA-treated tooth at 2 weeks, 1 month, and 5 months, respectively. There were three additional clinical failures after 58 and 59 months. The clinical and radiographic findings of these patients are shown in Table 4. The failed cases were treated with root canal treatment. The reasons for failures of the initial cases were either spontaneous pain or sensitivity to percussion, while that for failures of the additional cases, evident after long periods of observation, was pulp necrosis with apical periodontitis.

**Figure 3:**
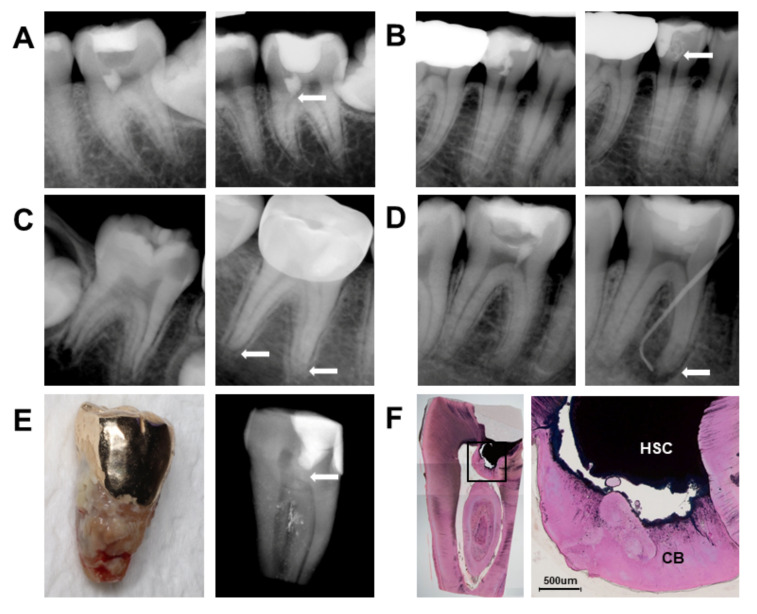
Radiographic and histologic evaluation of partial pulpotomy with three hydraulic silicate cements: (**A**) 17 Y, F, #36, OrthoMTA, 78 months follow-up: Calcific barrier formation (white arrow) with no clinical symptom. (**B**) 29 Y, F, #45, ProRoot MTA, 61 months follow-up; MTA has remarkably washed out (white arrow) with no clinical symptom and thin calcific barrier formation. (**C**) 6 Y, M, #46, OrthoMTA, 64 months follow-up: Apexogenesis is seen (white arrow). (**D**) 22 Y, M, #46, RetroMTA, 59 months follow-up: Increased periapical radiolucency with sinus tract formation (white arrow). (**E**) 49 Y, M, #37, ProRoot MTA, 78 months follow-up: Extracted tooth due to advanced periodontitis. The radiograph shows a thick calcific barrier formed without canal calcification (white arrow). (**F**) A histologic image of (**E**)—low magnification (left) and high magnification (right, 40×) images shows continuous calcific barrier under HSC. HSC: hydraulic silicate cement; CB: calcific barrier.

On radiographic analysis, a calcified barrier was distinguished in 75% of teeth, except for in cases not visible by the final restoration (Figure 3A). No noticeable canal calcification was found compared to neighboring teeth or contralateral teeth. A few cases showed wash-out of the capped MTA material, but we were unable to correlate this with treatment failure (Figure 3B). For all immature permanent teeth, apexogenesis (apex closure) was performed (Figure 3C). One failure case treated with RetroMTA showed increasing periapical radiolucency (Figure 3D). One case treated with ProRoot MTA was attributed to follow-up drop-out, being extracted due to advanced periodontitis (Figure 3E). In the results of the radiographic and histologic evaluation of teeth, a thick, poreless dentinal bridge formation was evident (Figure 3F).

## 4. Discussion

There are limited published articles comparing the long-term effectiveness of various HSCs in partial pulpotomy. Therefore, this study presented the long-term properties and biocompatibility of treatment of partial pulpotomy compared to our previous short-term randomized controlled trial. After 4 years, partial pulpotomy with HSCs had a favorable success rate (89.3%). Compared to the results at 12 months, the 48–78 month success rate of partial pulpotomy using ProRoot MTA decreased by 6% (96.0%→90.0%), and those of OrthoMTA and RetroMTA decreased by 4.3% (92.8%→88.5%) and 6.6% (96%→89.4%), respectively [20]. The rate of long-term success was similar or slightly higher than in previous studies of partial pulpotomy with HSCs: 93%, 34.8 months follow-up [3]; 85%, 24 months follow-up [18]; and 79%, 12–26 months follow-up [19]. This study presents a novel long-term study which sustained high success rates of partial pulpotomy with various HSCs, even though there was no statistical difference among the three groups.

On a long-term basis, pulp-treated teeth were recommended to be followed for longer periods to evaluate success rates. The follow-up period in this study was a mean 62.7 months. In the previous study, early failures after vital pulp therapy with calcium hydroxide were observed. Matzuo et al. [24] reported that 3 months was adequate to determine a tentative prognosis, and 21 months was recommended as a follow-up period to evaluate post-operative prognosis for direct pulp capping. Mejàre and Cvek [25] and Zilberman et al. [26] reported that failure of a pulp-capping procedure can often be detected in the first 10 days and 20 days after treatment. However, there is no accurate established timing considered as success for partial pulpotomy, as this procedure presents a high success rate over different follow-up periods. Thus, we performed a long-term follow-up study to evaluate late failure, in contrast to the previous study, in which we evaluated early failure. Based on a recent report, this is the longest follow-up study of a randomized controlled trial on HSC partial pulpotomy.

The immediate/early failures occurred in the initial 5 months after treatment in four cases, within 2 weeks in two cases, within 1 month in one case, and within 5 months in one case. The reason for early failures was commonly expressed as spontaneous pain, which is associated with the inflammatory process [27]. As teeth were restored with final restorations, the reason for early failure could be that the treatment itself served as a possible pathway for bacterial contamination. Interestingly, three teeth failed after 58 and 59 months. Such delayed/late failures arose because no new hard tissue was formed or because the newly formed hard tissue did not act as a protective barrier against bacterial microleakage [28]. It can be assumed that bacterial leakage could play a role in pulp necrosis and periapical involvement in more extended follow-up periods.

No significant difference was observed between the three HSCs in this study; however, clinicians should pay attention to the physical and chemical properties of OrthoMTA and RetroMTA. Initial failure occurred within 2 weeks in the cases with OrthoMTA, which may have been due to the possible cytotoxicity of the raw material itself. In a cell viability assay, OrthoMTA has been shown to be significantly more cytotoxic than ProRoot MTA [29,30]. Further, heavy metals in HSCs are of concern as they come into direct contact with the pulp and hard tissues. As such, OrthoMTA contains fewer heavy metal components (Cd, Cu, Fe, Mn, and Ni) than ProRoot MTA, except for Zn [11]. Nonetheless, ProRoot MTA and OrthoMTA have been reported to meet the ISO regulations regarding the safety limits [22] and are considered safe biomaterials for long-term clinical use. Furthermore, in an ultraviolet spectrophotometric analysis, RetroMTA showed the highest dye-absorbance values when compared with ProRoot MTA [31]. The difference in sealing ability can have an influence on the furcation repair materials; however, in this study, no significant difference was observed between the two materials. Moreover, in an in vitro cytotoxicity assessment, RetroMTA reportedly showed better biocompatibility compared with the calcium-enriched mixture, Angelus MTA [32]. However, in the histologic analysis after partial pulpotomy in permanent teeth, RetroMTA showed pulp disorganization, an absence of inflammation, and discontinuous mineralization, which may represent a potential drawback [33].

Although we evaluated several potential prognostic factors such as age, arch, tooth type, type of HSC, cause of exposure, apex status, distance to exposure, site of exposure, and final restoration, the results for each clinically significant variable indicated that there were no significant factors affecting survival rate. This finding is in accordance with a meta-analysis study of partial pulpotomy which reported that the final restoration, pulp-capping material, apex closure, and age of the patient did not affect treatment results [28]. In this meta-regression analysis, the only variable significantly associated with success rate at 1-year follow-up was preoperative pulp status [28]. The study was a randomized clinical study conducted by screening patients suitable for the indication of partial pulpotomy. Thus, inadequate pulp status in this treatment was already considered to be excluded. Though all failed cases had closed apex state, neither the patient’s age nor root apex closure status affected the prognosis of partial pulpotomy. In partial pulpotomy, teeth with an open apex are considered to have a higher potential for healing, and the remaining pulp is relatively healthy with regeneration abilities [34]. However, most studies reported that teeth with a closed apex had no significant differences when compared to teeth with an open apex [18,35]. As it is difficult to determine the criteria to differentiate between younger and older individuals, this study formed age groups based on the average value of “30 years”, considering that the study subjects were aged between 6 and 68 years and that the average age was 29.3 ± 14.83 years. From the results of this study, we cannot conclude that the success rate was higher in young patients, because the seven failed cases occurred in patients in their 20s and 30s. However, age did not affect the success rate in this study significantly (*p* = 1), and patients in the wide age range of 6 to 68 years were treated successfully with partial pulpotomy. Thus, our results show that preoperative pulpal condition and selection of patients should be considered while determining the success of HSC partial pulpotomy.

Despite previous findings of canal calcification after pulpotomy, no cases showed obvious canal calcification after 4 years of partial pulpotomy. We made comparisons with adjacent teeth, contralateral teeth, or canals in distances with the pulp capping area within the same tooth. In cases where canal spaces were noticeably decreased, other teeth showed similar changes, implying that canal space narrowing is not from the partial pulpotomy. Coronal pulpotomy often results in complete obliteration of the root canals, which could be considered an unacceptable outcome. Because canal calcification leads to diminished blood supply and pulp necrosis, it should be assessed carefully [35]. In addition, development of a calcified barrier with other vital signs shows a favorable response to vital pulp therapy. In radiographic findings, 75% of total cases had calcific barriers at 3–64 months (mean 21.3), with no statistical differences in calcified barrier formation among the three HSC materials.

This study was subject to several limitations that should be addressed. First, the ratio of loss to follow-up in this study was more than 20%. A minimum rate of >80% is required for a high strength level of evidence [36]. Although we attempted to recall the patients, most of them refused recall visits for asymptomatic teeth. It might be irrational to consider the asymptomatic patients who did not return for recall visits as successful cases. However, according to the results of our study, 5 of the 7 patients with failed cases showed subjective symptoms. Additionally, considering the success rate of partial pulpotomy in the patients who completed the follow-up in the previous study and in this study, it may not be an unreasonable assumption that the patients dropping out of this study due to the absence of subjective symptoms had little effect on the results. Moreover, this study used an intention-to-treat (ITT) analysis to reduce major compliance on randomized controlled trials [37]. This helped to avoid overoptimistic estimates of efficacy of the intervention by ignoring non-compliance, protocol deviations, withdrawal, and/or any unfavorable event occurring after randomization in clinical practice [38]. Second, a multivariable statistical analysis for these time-censored data would have been a survival analysis with a proportional hazards regression model. However, as the incidence rates of failure were considerably low in this study, the validity of such an analysis would have been compromised. This study is meaningful because it is the first attempt to evaluate the long-term prognosis of partial pulpotomy using different HSCs. It can provide a reliable basis for further research with less follow-up loss.

## Figures and Tables

**Table 1:** Composition of ProRoot MTA, OrthoMTA and RetroMTA.

Material	Composition
ProRoot MTA	Tricalcium silicate, (CaO)_3_·SiO_2_
	Dicalcium silicate, (CaO)_2_·SiO_2_
	Tricalcium aluminate, (CaO)_3_·Al_2_O_3_
	Tetracalcium aluminoferrite, (CaO)_4_·Al_2_O_3_·Fe_2_O_3_
	Gypsum, CaSO_4_·2H_2_O
	Free calcium oxide, CaO
	Bismuth oxide, Bi_2_O_3_
OrthoMTA	Tricalcium silicate, (CaO)_3_·SiO_2_
	Dicalcium silicate, (CaO)_2_·SiO_2_
	Tricalcium aluminate, (CaO)_3_·Al_2_O_3_
	Tetracalcium aluminoferrite, (CaO)_4_·Al_2_O_3_·Fe_2_O_3_
	Free calcium oxide, CaO
	Bismuth oxide, Bi_2_O_3_
RetroMTA	Calcium carbonate (CaCO_3_)
	Silicon dioxide (SiO_2_)
	Aluminum oxide (Al_2_O_3_)
	Calcium zirconia complex

**Table 2:** Descriptive statistics of partial pulpotomy with hydraulic silicate cements (HSCs) in long-term (48–78 months) follow-up group (*n* = 65).

Variable	Censored (*n*, %)	Failure (*n*, %)	Total	*p* Value ^a^
Age (y)				
<30	36 (87.8)	5 (12.2)	41	
≥30	22 (91.7)	2 (8.3)	24	1
Arch				
Lower	25 (92.6)	2 (7.4)	27	
Upper	33 (86.8)	5 (13.2)	38	0.69
Tooth type				
Molar	33 (89.2)	4 (10.8)	37	
Premolar	25 (89.3)	3 (10.7)	28	1
Type of HSC ^a^				
ProRoot MTA	18 (90.0)	2 (10.0)	20	
OrthoMTA	18 (85.7)	3 (14.3)	21	
RetroMTA	22 (91.7)	2 (8.3)	24	0.88
Cause of exposure				
Caries	54 (90.0)	6 (10.0)	60	
Trauma	4 (80.0)	1 (20.0)	5	0.45
Apex status				
Closed	49 (87.5)	7 (12.5)	56	
Open	9 (100.0)	0 (0.0)	9	0.58
Distance to exposure (mm) ^b^				
>0.5	36 (90.0)	4 (10.0)	40	
≤0.5	22 (88.0)	3 (12.0)	25	1
Site of exposure				
Occlusal	19 (90.5)	2 (9.5)	21	
Axial	39 (88.6)	5 (11.4)	44	1
Final restoration ^c^				
Indirect	27 (80.0)	3 (10.0)	30	
Direct	31 (88.6)	4 (11.4)	35	1

^a^ Fisher’s exact test was used to determine associations between categorical variables. ^b^ Distance between cavity and pulp seen in initial periapical radiograph. ^c^ Indirect, crown, onlay, and inlay restoration; direct, resin restoration.

**Table 3:** Univariate Cox regression analysis according to the potential clinical variables (*n* = 104).

Variable	Censored (*n*, %)	Failure (*n*, %)	Total	*p* Value	Hazard Ratio (95% CI)
Age (y)					
<30	59 (92.2)	5 (7.8)	64		1
≥30	38 (95.0)	2 (5.0)	40	0.32	0.31 (0.03–3.14)
Arch					
Lower	43 (95.6)	2 (4.4)	45		1
Upper	54 (91.5)	5 (8.5)	59	0.33	2.62 (0.38–18.04)
Tooth type					
Molar	56 (93.3)	4 (6.7)	60		1
Premolar	41 (93.2)	3 (6.8)	44	0.68	0.67 (0.1–4.46)
Type of HSC ^a^					
ProRoot MTA	31 (93.9)	2 (6.1)	33		1
OrthoMTA	33 (91.7)	3 (8.3)	36	0.88	0.85 (0.1–7.26)
RetroMTA	33 (94.3)	2 (5.7)	35	0.97	1.04 (0.1–10.69)
Cause of exposure					
Caries	91 (93.8)	6 (6.2)	97		1
Trauma	6 (85.7)	1 (14.3)	7	0.66	1.91 (0.11–34.56)
Apex status					
Closed	81 (92.0)	7 (8.0)	88		1
Open	16 (100.0)	0 (0.0)	16	1	0.0000001 ^b^
Distance to exposure (mm) ^c^					
>0.5	59 (93.7)	4 (6.3)	63		1
≤0.5	38 (92.7)	3 (7.3)	41	0.37	2.16 (0.4–11.56)
Site of exposure					
Occlusal	32 (94.1)	2 (5.9)	34		1
Axial	65 (92.9)	5 (7.1)	70	0.88	1.19 (0.13–11.0)
Final restoration ^d^					
Indirect	46 (93.9)	3 (6.1)	49		1
Direct	51 (92.7)	4 (7.3)	55	0.75	0.75 (0.12–4.54)

^a^ HSC—hydraulic silicate cement. The hazard ratios were calculated between ProRoot MTA and other two materials. ^b^ Undefined (95% confidence interval (CI) = 0 to infinity). ^c^ Distance between cavity and pulp seen in initial periapical radiograph. ^d^ Indirect, crown, onlay, and inlay restoration; direct, resin restoration.

**Table 4:** Characteristics of total failed cases in follow-up of partial pulpotomy with HSCs.

Age	Tooth Type	Apex	Exposure Type	Site of Exposure	HSCs Material	Decision of Failure	Reason of Failure
25	Premolar	Closed	Trauma	Axial	OrthoMTA	2 Weeks	Spontaneous pain
25	Premolar	Closed	Caries	Axial	OrthoMTA	2 Weeks	Spontaneous pain
26	Molar	Closed	Caries	Occlusal	ProRoot MTA	1 Month	Spontaneous pain and sensitivity to percussion
28	Molar	Closed	Caries	Axial	RetroMTA	5 Months	Sinus tract formation and periapical radiolucency
22	Molar	Closed	Caries	Occlusal	RetroMTA	58 Months	Sinus tract formation and periapical radiolucency
39	Molar	Closed	Caries	Axial	OrthoMTA	59 Months	Periapical radiolucency
37	Premolar	Closed	Caries	Axial	ProRoot MTA	59 Months	Periapical radiolucency

## Data Availability

Data is contained within the article.
